# ERAS (enhanced recovery after surgery) protocol improves recovery in surgical management of pediatric non-cirrhotic portal hypertension: evaluating results of pre and post-ERAS implementation

**DOI:** 10.1007/s00383-026-06505-4

**Published:** 2026-06-21

**Authors:** Samir Hasan, Ülgen Çeltik, Orkan Ergün

**Affiliations:** Department of Pediatric Surgery, Faculty of Medicine, Ege University, Izmir, Turkey

**Keywords:** Portal hypertension, Rex shunt, Distal splenorenal shunt, ERAS, Child

## Abstract

**Purpose:**

Standardized perioperative pathways for pediatric non-cirrhotic portal hypertension (PHT) requiring complex portosystemic shunt surgery remain scarce. Enhanced Recovery After Surgery (ERAS) protocols aim to reduce surgical stress and optimize postoperative recovery. This study evaluates the impact of ERAS implementation on outcomes in children undergoing shunt procedures for PHT.

**Methods:**

A retrospective cohort study was conducted over 18 years. Patients were divided into two groups: Group I (pre-ERAS,2006–2016) and Group II (post-ERAS,2016–2024). Demographics, surgical type, time to oral feeding, mobilization, drain usage, ICU stay, hospital stay, and 30-day readmissions were analyzed.

**Results:**

A total of 103 patients (M/F: 50/53; mean age 8.4 ± 5 years) underwent shunt surgery. Fifty-one were treated before and 52 after ERAS implementation. Distal splenorenal shunt was most common (*n* = 74), followed by Rex shunt (*n* = 20) and other procedures (*n* = 9). ERAS was associated with significantly earlier oral feeding (1.1 vs.1.6 days, *p* = 0.007), earlier mobilization (1.15 vs.1.6 days, *p* = 0.046), markedly reduced drain use (1.9% vs. 23.5%, *p* = 0.001), shorter ICU stay (1.48 vs.1.9 days, *p* = 0.03), and nearly halved hospital stay (4.6 vs.8.5 days, *p* = 0.001). Thirty-day readmissions were similar.

**Conclusion:**

ERAS implementation in pediatric PHT surgery is feasible, safe, and associated with substantially improved recovery and standardized perioperative care.

## Background

Pediatric non-cirrhotic portal hypertension (PHT), mainly due to portal vein thrombosis, is a rare clinical problem [1–3]. Surgical management is required when optimal conservative management fails and generally includes portosystemic shunt procedures to control variceal bleeding and hypersplenism [4]. Portosystemic shunt surgery in children is particularly infrequent and challenging. These procedures represent some of the most technically demanding interventions in pediatric hepatobiliary surgery and are typically performed in a limited number of specialized centers [5]. The rarity and complexity of such cases imply that few pediatric surgeons perform such procedures regularly, resulting in substantial variability in perioperative management across centers. Concerns regarding postoperative complications often translate into prolonged fasting, extended drain duration, delayed mobilization, conservative nutritional advancement, excessive workup, overall longer hospitalization, and increased costs, which may in turn increase postsurgical stress and affect recovery [6]. The absence of standardized care pathways also leads to variable outcomes [7].

Recently, surgical care has increasingly focused on reducing physiological stress and improving recovery [8]. The Enhanced Recovery After Surgery (ERAS) protocols, introduced in the late 1990s, offer a multidisciplinary, evidence-based approach to optimize perioperative care and support faster functional recovery [9]. Applying ERAS principles to complex procedures such as shunt surgery for PHT may help standardize care, shorten hospitalization, reduce complications, and enhance overall recovery without compromising safety [10–12].

To date however, no published study has specifically assessed the application of ERAS principles in pediatric PHT surgery and portosystemic shunt procedures. This gap in the literature necessitates the need for standardized, evidence-based perioperative pathways in a field where practice patterns remain highly variable.

This study evaluates the feasibility and positive effects of implementing the ERAS protocol in complex surgical procedures such as portosystemic shunt surgery in children. By sharing our institutional practice, we therefore aim to contribute to the development of more consistent, optimized care strategies for this rare but challenging pediatric population.

## Methods

### Ethical approval

This study was conducted in accordance with the ethical principles of the Declaration of Helsinki and approved by the Medical Research Ethics Committee of Ege University Faculty of Medicine (Approval number: 25-10T/17). Written informed consent was obtained from the legal guardians of all pediatric patients included in this study. The authors affirm that all methods were performed in accordance with the relevant guidelines and regulations.

### Patient management

In our institution, children with PHT are managed through a structured perioperative pathway that begins with a comprehensive clinical and radiological evaluation consisting of endoscopy, Doppler ultrasonography, Computerized Angio-Tomography (CT-Angio)/Magnetic Resonance angiography, thrombosis panel, complete blood counts and biochemistry, followed by individualized selection of the appropriate surgical strategy based on vascular anatomy and portal flow dynamics.

**Fig. 1:**
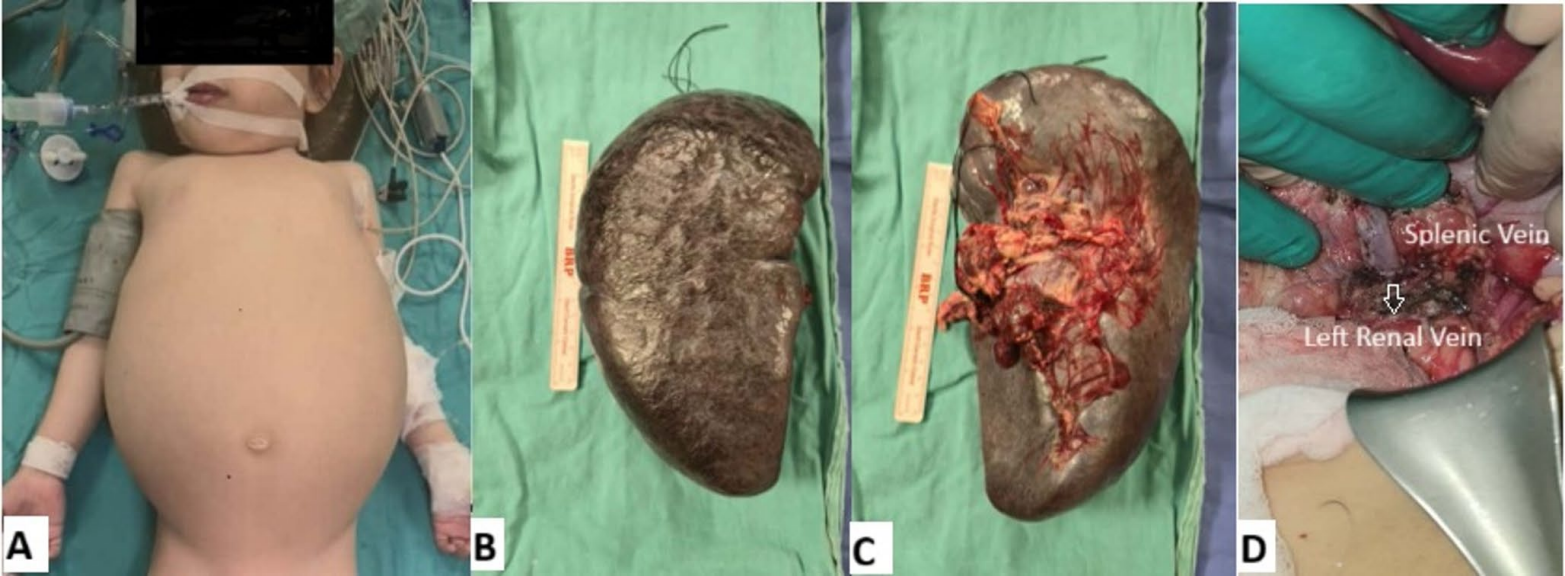
**A** Preoperative abdominal view, **B** Lateral surface of the spleen, **C** Medial surface of the spleen, **D** Intraoperative view of proximal splenorenal shunt

Patients with portal vein thrombosis who are found to have patent intrahepatic portal branches, especially on the left portal vein/Rex site, are considered for Rex Shunt (Mesenteric-Left Portal By-pass), while those who have no favorable or phlebosclerotic intrahepatic portal vein branches that seem to be incompatible with carrying the portal flow gradient are considered for the splenorenal shunt procedure. Distal splenorenal shunt is the procedure of choice unless the patient has a huge spleen impairing the child’s comfort and mobility; in this case, proximal splenorenal shunt with splenectomy was performed in very rare and selected cases (Fig. 1). All operations were performed by an experienced pediatric hepatobiliary surgeon (OE).

In 2016, a comprehensive ERAS protocol was integrated into the perioperative management of PHT patients (Table 1). Our protocol includes detailed pre- and postoperative family counseling, shortened fasting without routine bowel preparation, and initiation of low-molecular-weight heparin prophylaxis before surgery. Intraoperatively, multimodal anesthesia with reduced opioid use, maintenance of normothermia, balanced fluid therapy, and highly selective use of drains or nasogastric tubes are practiced. Nasogastric tube is avoided in the majority of cases, especially because of the possibility of injury to esophageal varices during insertion of the tube. A nasogastric tube is only placed if the stomach is overdistended, impairing surgical exposure; in this case, a well-lubricated NG tube is slowly advanced by the anesthesiologist under the manual control of the surgeon. Perioperative transfusion of blood products is not our regular practice and is rarely indicated.

**Table 1:** ERAS Protocol for pediatric portal hypertension

	ERAS group	Non-ERAS (conventional) group
Preoperative education, counselling, and psychological support	Yes	No
Mechanical bowel preparation	No	Yes
Preoperative fasting period	5–6 h (age dependent)	Overnight 9–12 h
Timing of NG and Foley catheter insertion	After anesthesia (NG tube avoided unless strictly necessary)	Before entering the operating room
Fluid replacement therapy during surgery	Standardized	Based on the anesthesiologist’s experience
Analgesic management during surgery	Standardized	No standardized
Timing of NG tube and urinary catheter removal	First day after surgery	Urinary catheter: 2.day Gastric tube: After passing gas (2 or 3 days)
Timing of abdominal drain removal	No drain	Postoperative day 2 or 3
Timing of postoperative mobilization	Within first 12 h	Unspecified
Timing of first postoperative feeding	First day after surgery – following Doppler confirmation of shunt patency	Postoperative day 2 or 3

Postoperatively, Doppler ultrasonography is routinely performed on postoperative day 1 to verify shunt patency, after which oral feeding is resumed; initially, fluid administration rapidly advances to normal enteral feedings as per tolerance of the patient. Patients are initially kept in the intensive care unit and transferred to the clinic after 12 h of monitoring. Early mobilization is encouraged on the day of surgery, and discharge typically occurs on postoperative day 2 or 3. Patients treated before the implementation of ERAS received traditional perioperative care without a standardized protocol. Long term follow-up protocol is not within the scope of this manuscript.

### Study design

This study was designed as a retrospective cohort analysis conducted at Ege University Faculty of Medicine, Department of Pediatric Surgery, and included an 18-year period from January 2006 to December 2024, during which portosystemic shunt procedures were performed for the treatment of pediatric portal hypertension.

### Inclusion and exclusion criteria

#### Inclusion criteria

- Pediatric patients aged under 18 years.
- Diagnosed with non-cirrhotic portal hypertension.
- Underwent portosystemic shunt surgery (Rex shunt, distal or proximal splenorenal shunt).
- Complete perioperative data available.
- Surgery performed between January 2006 and December 2024.

#### Exclusion criteria

- Patients with cirrhotic portal hypertension.
- Patients who underwent non-shunt procedures.
- Incomplete clinical records or follow-up data.

To assess the impact of ERAS implementation, patients were categorized into two cohorts based on the year of operation: Group I (Pre-ERAS), comprising children operated between 2006 and 2016 under conventional perioperative care, and Group II (ERAS), including those operated between 2016 and 2024 following the formal introduction of ERAS principles. All relevant information was obtained from electronic medical records, operative reports, intensive care charts, and follow-up documentation. Collected variables included demographic characteristics, type of shunt procedure, intraoperative drain use, time to initiation of oral feeding, time to discontinuation of intravenous fluid support, time to first mobilization, duration of intensive care unit stay, overall length of hospital stay, and 30-day readmission.

### Statistical analysis

An a priori biostatistical power analysis was conducted using G*Power software (version 3.1.9.4) to determine the required sample size for a two-tailed independent samples t-test. Assuming an effect size (Cohen’s d = 0.8), a significance level of α = 0.05, and a power of 90%, the analysis revealed that 34 patients per group (total *n* = 68) would be sufficient to detect a statistically significant difference in postoperative hospital stay duration, which was designated as the primary outcome measure.

Statistical analyses were performed using IBM SPSS Statistics version 27.0 (IBM Corp., Armonk, NY, USA). Continuous variables were expressed as mean ± standard deviation (SD) or median with range, as appropriate. Categorical variables were presented as frequencies and percentages. Comparisons between groups were made using the Student’s t-test or Mann–Whitney U test for continuous variables, and the Chi-square test for categorical variables. A two-tailed p-value < 0.05 was considered statistically significant.

## Results

A total of 103 children underwent portosystemic shunt surgery for PHT between 2006 and 2024, including 50 males and 53 females with a mean age of 8.4 ± 5 years. Group I consisted of 51 patients, whereas Group II included 52 patients. Table 2 summarizes the results of pre- and post-ERAS protocols.

Across the entire cohort, the distal splenorenal shunt was the most frequently performed procedure (74 patients). The Rex shunt was performed in a total of 20 children, with a markedly higher proportion in the pre-ERAS period (16 vs. 4). Other portosystemic procedures—including mesocaval or unconventional shunts and coronary vein ligation—were carried out in 9 patients overall.

After the implementation of ERAS, drain usage decreased significantly, dropping from 23.5% in Group I to only 1.9% in Group II (*p* = 0.001), demonstrating a clear shift toward avoiding unnecessary drain placement.

Early mobilization was achieved significantly sooner in Group II when compared with Group I (1.15 vs. 1.6 days, *p* = 0.046). The initiation of oral feeding occurred significantly earlier in Group II (mean 1.1 vs. 1.6 days, *p* = 0.007). ICU stay was significantly shorter in Group II than in Group I (1.48 vs. 1.9 days, *p* = 0.03), Similarly, total

hospital length of stay was nearly halved in Group II compared with Group I (4.6 vs. 8.5 days, *p* = 0.001). Notably,

30-day readmission rates did not differ significantly between the two groups.

**Table 2:** Comparative results of Pre-and Post-ERAS implementation in PHT patients

Parameters	2006–2016 (ERAS-) (*n* = 51)	2016–2024 (ERAS+) (*n* = 52)	*p*-value
**Surgical techniques**			
REX shunt	16	4	
Distal splenorenal shunt	28	46	
Other	7	2	
Drain usage	12	1	0.001*
Time to start oral feeding (days)	1.6(1–7)	1.1(1–3)	0.007*
Time to discontinue IV fluid support (days)	1.8(1–8)	1.3(1–6)	0.009*
Time to start postoperative mobilization (days)	1.6(1–7)	1.15(1–4)	0.046*
ICU stay duration (days)	1.9(1–9)	1.48(1–4)	0.030*
Hospital stay duration (days)	8.5(4–39)	4.6(2–16)	0.001*
30-day readmission	8	5	0.35

*p-values < 0.05 were considered statistically significant

## Discussion

Despite remarkable progress in pediatric anesthesia and surgical techniques, postoperative complications and prolonged recovery remain major challenges, especially in complex conditions. To overcome these difficulties, Enhanced Recovery After Surgery (ERAS) protocols have emerged as a modern, evidence-based approach designed to minimize surgical stress, support faster physiological recovery, and improve overall patient outcomes [10, 11, 13].

Originally developed for adult colorectal surgery, ERAS principles have now expanded into pediatric practice, where their potential impact may be even greater [14]. Children experience unique and often amplified physiological stress responses to surgery, making optimized perioperative care particularly crucial [15].

This study demonstrates that the implementation of an Enhanced Recovery After Surgery (ERAS) protocol in pediatric PHT surgery is both feasible and clinically beneficial. Although ERAS pathways have been widely adopted in adult surgical practice and increasingly in routine pediatric procedures, their application to complex hepatobiliary operations—particularly shunt surgeries for portal hypertension—has not been previously evaluated. Our findings provide some of the first evidence that structured perioperative optimization can meaningfully improve recovery trajectories in this highly specialized patient population.

A key observation of this study was the marked reduction in surgical drain usage after ERAS implementation, decreasing from 23.5% in Group I to only 1.9% in Group II. This change reflects an intentional shift away from routine drainage toward selective, indication-based use. Avoiding unnecessary drains is known to reduce postoperative discomfort, facilitate early mobilization, and lower the risk of infection, each of which may contribute to smoother postoperative recovery in children undergoing major abdominal surgery [6, 16].

The improvements in functional recovery parameters—including earlier initiation of oral feeding and faster postoperative mobilization—represent central advantages of ERAS protocols. Time to oral intake improved significantly in the ERAS cohort, supporting the safety and utility of early enteral nutrition in this context. Early feeding is associated with improved gut motility, reduced catabolism, and enhanced immunologic integrity, which are particularly valuable in children who often have preexisting malnutrition secondary to chronic portal hypertension [17]. Similarly, mobilization achieved a median of half a day earlier in Group II, a seemingly modest but clinically important difference given its role in reducing pulmonary and thromboembolic complications [18].

Most remarkable finding was the substantial reduction in hospital length of stay, which was nearly halved in Group II compared with Group I. Shortened hospitalization is a well-recognized downstream effect of ERAS pathways and carries multiple advantages, including reduced healthcare costs, decreased nosocomial risk exposure, and greater comfort and convenience for families [19–21]. The concurrent decrease in ICU duration further supports the notion that ERAS accelerates physiological stabilization after complex hepatobiliary surgery. Importantly, the observed improvements in postoperative recovery did not come at the expense of patient safety, as 30-day readmission rates were comparable between the two groups [21, 22].

Another important dimension is the economic impact of ERAS implementation. Morin et al. specifically reported that the adoption of an ERAS protocol in pediatric cranial vault reconstruction led to a significant reduction in hospital stay and associated healthcare costs, demonstrating the financial advantages of enhanced recovery pathways. They also emphasized that shorter ICU stays contributed to overall resource savings, further underlining the economic sustainability of ERAS in pediatric surgery [23]. These findings are in accordance with our observation that halving the hospital stay and reducing ICU utilization can yield not only clinical but also economic benefits in complex pediatric procedures.

In pediatric portal hypertension where surgical interventions such as shunt procedures are associated with significant postoperative risk, implementing ERAS strategies offers a promising opportunity to reduce complications, enhance comfort, shorten hospital stays, and support safer, smoother recovery aiming to demonstrate how structured, multidisciplinary perioperative care can transform outcomes in this vulnerable population.

## Limitations

This study has several limitations. Its retrospective design carries an inherent risk of selection and information bias, and the evolution of surgical expertise over an 18-year period may have influenced outcomes independent of ERAS implementation. Additionally, the single-center setting, although advantageous for maintaining consistency in surgical technique and perioperative protocols, may limit the generalizability of the findings to institutions with different levels of experience or resource availability. Further multicenter prospective studies are needed to confirm these results and to refine ERAS strategies specific to pediatric portal hypertension surgery.

## Conclusion

This study represents the first report to evaluate the application of ERAS principles in pediatric PHT surgery, demonstrating that even the most complex shunt procedures can safely benefit from a structured, evidence-based recovery pathway. Despite the technical challenges inherent to these operations, our findings show that ERAS markedly improves postoperative recovery, reduces resource utilization, and standardizes care without increasing complications. These results highlight an essential message for the pediatric surgical community: complexity should not discourage the adoption of ERAS.

## Data Availability

No datasets were generated or analysed during the current study.
